# Ignoring Spatial and Spatiotemporal Dependence in the Disturbances Can Make Black Swans Appear Grey

**DOI:** 10.1007/s11146-021-09836-2

**Published:** 2021-03-31

**Authors:** R. Kelley Pace, Raffaella Calabrese

**Affiliations:** 1 LREC Endowed Chair of Real Estate, Department of Finance, Louisiana State University, Baton Rouge, LA USA; 2 Associate Professor in Data Science, Credit Research Centre, Business School University of Edinburgh, Edinburgh, Scotland

**Keywords:** Spatial, Spatial-temporal, Non-normal distributions, Kurtosis, Rare events, Automated valuation models, Housing prices

## Abstract

Automated valuation models (AVMs) are widely used by financial institutions to estimate the property value for a residential mortgage. The distribution of pricing errors obtained from AVMs generally show fat tails (Pender 2016; Demiroglu and James *Management Science, 64*(4), 1747–1760 2018). The extreme events on the tails are usually known as “black swans” (Taleb 2010) in finance and their existence complicates financial risk management, assessment, and regulation. We show via theory, Monte Carlo experiments, and an empirical example that a direct relation exists between non-normality of the pricing errors and goodness-of-fit of the house pricing models. Specifically, we provide an empirical example using US housing prices where we demonstrate an almost perfect linear relation between the estimated degrees-of-freedom for a Student’s *t* distribution and the goodness-of-fit of sophisticated evaluation models with spatial and spatialtemporal dependence.

## Introduction

Financial distress, such as experienced during the Great Recession and now as a result of the novel coronavirus, requires servicers and lenders to make decisions on new lending (refinancing or new purchases), forbearance, loan extensions, and foreclosure in the presence of a number of state laws and changing regulator guidelines. Most of these decisions require some estimate of collateral value. Financial institutions in the real estate market usually rely on experts, called appraisers, to estimate the value of the collateral. Because appraisers have substantial freedom in creating their estimate (Agarwal et al. 2015), the estimate is ultimately subjective. Automated valuation models (AVMs) provide an alternative approach (Pender 2016; Demiroglu and James 2018). An AVM^1^ is a computer program that can use property characteristics, past house prices, and neighboring comparable sales in a statistical model to value property.

The Great Recession raised questions about the accuracy of appraisals and AVMs. Using a large sample of nonagency securitized loans originated between 2002 and 2007, Griffin and Maturana (2016) show that AVMs are more accurate than most appraisers as 44.9% of residential properties show appraisal values that are 5% higher than an AVM. Agarwal et al. (2015) examine the appraisal bias for residential refinance transactions. Particularly, they compute the valuation bias as the difference between the appraisal of the refinance transaction and the consecutive purchase price. On a US national sample of conforming loans, the authors find that the appraisal bias for residential refinance transactions is more than 5%.

Ding and Nakamura (2015) compare the appraised value of the property with the agreed contract price between the buyer and the seller to analyse the impact of the Home Valuation Code of Conduct (HVCC) in 2009 on the appraisal practice. They find that appraisal valuations are on average higher than the contract price both before and after the financial crisis, even if the HVCC has led to a decrease of the over-estimate of the property value. For the accuracy of AVMs, Demiroglu and James (2018) provide evidence that AVMs show high pricing errors of between 12% and 15% of the actual sale price for a median quality home. The variability is even higher for properties below median quality. The authors also show that high pricing errors can be the cause of an apparent appraisal bias even when the AVMs are unbiased.

Krugery and Maturanaz (2020) analyze appraisals and AVMs on a large sample of US non-agency securitized loans originated between 2001 and 2007. They highlighted that the potential sources of the errors for AVMs and appraisals are different. AVM errors are statistical errors or due to model miscalibration, whereas moral hazard could potentially generate the appraisal errors. The authors obtain empirical evidence of high appraisals relative to AVM valuations over time and across different types of loans. Specifically, appraisals are almost 5% higher (on average) than AVM valuations and appraisals exceed AVM valuations 60% of the time.

The main contribution of this paper is to explain why AVMs may yield accurate predictions with highly non-normal errors. Specifically, this manuscript examines the relation between increasing the goodness-of-fit of housing models and the need to model the house price distribution of the disturbances. AVMs mainly rely on house price models that often show highly non-normal errors (Young and Graff 1996; Gu 2002; Pontines 2010; Schindler 2013) and high levels of spatial dependence (Bourassa et al. 2007; Liu 2013; Pace 1997; Osland 2010). Using both Monte Carlo simulations and empirical data, we show that addressing spatial or spatiotemporal dependence could lead to an increased precision of the house price prediction which, in turn, could unmask non-normal disturbances. Consequently, more sophisticated approaches, such as spatial regression models (LeSage and Pace 2009), can lead to non-normal disturbances with a lower magnitude.

Leading AVM companies are Zillow, Collateral Analytics and CoreLogic, among others. For example, Griffin and Maturana (2016) analyse data provided by Collateral Analytics. Although all of the providers of AVMs give some statistics on their accuracy, we will use Zillow as a motivational example as Zillow provides the most visible automated valuation model available to the general public with over 100 million price estimates (known as “Z-estimates”) provided on their website at no pecuniary charge.

We assume that Zillow has adequately modeled house prices using accurate explanatory variables as well as handling various forms of temporal, spatial, and spatiotemporal dependence known to affect house prices (Aquaro et al. 2021; Can and Megbolugbe 1997; Pace 1997).^2^ Therefore, the Z-estimate residuals should convey some information about the underlying distribution of the disturbances. Zillow reports nationally that their models have a median absolute percentage error of 4.5%. Moreover, 89.7% of the Z-estimates exhibit absolute percentage errors of 20% or less.

Under the assumption that the percentage errors are symmetric, a Student *t* distribution would provide a common way of capturing the kurtosis that empirical residuals often display (Pontines 2010). Fitting a Student *t* distribution to the Zillow reported statistics results in an estimate of 1.37 degrees-of-freedom, which means that the variance and kurtosis are infinite. Compare this to a Cauchy distribution (Student *t* with 1 degree-of-freedom) which for a median absolute percentage error of 4.5% would have 86% of the absolute residuals within 20% (as opposed to the 89.7% in Zillow). If the true innovations in house price models follow a Cauchy-like distribution, this means that large errors are not particularly rare and this has implications for financial risk management, assessment, and regulation. In the context of finance, such rare events have been labeled “black swans” (Taleb 2010).

In this paper we show both theoretically and through Monte Carlo simulations that disturbances in house price models which ignore spatial-temporal dependence can appear more normally distributed. Using US census data from 2000 we obtain a strong linear relationship between the accuracy of a house price model and the tail heaviness (indicated by the level of leptokurtosis) of the residuals. We can sum up that assuming non-normal distribution for the disturbances could provide at least one way to address the issue of “black swans.” In contrast, simple models that ignore spatial or spatiotemporal dependence may lead to “normal” residuals that mask non-normality. In other words, not modeling spatial and spatiotemporal dependence can make “black swans” appear grey.

We begin with “The Zillow Example” where we analyze the kurtosis of the residuals in the Z-estimates. “The Kurtosis in Regression Models with Spatial and Temporal Dependence” documents the kurtosis present in the residuals from house price models and how this makes the risk assessment process more difficult. We motivate why residuals from non-spatial models may mask non-normality and provide both theoretical and Monte Carlo evidence to support this statement. In “An Empirical Example” we set forth an example based on housing data, where we show a very strong empirical relation between the house price accuracy and the leptokurtosis of the residuals. We finish in “Conclusion” with a summary of the key findings and discuss the implications of this research for housing models and other areas of application.

## The Zillow Example

In this section we analyse the kurtosis of the residuals in the Zillow house price model. We use the data published on Zillow’s website on September 30, 2016. We assume that the error random variable *u* is a Student *t* with *ν* degrees-of-freedom so that fat tails can be obtained (Pontines 2010). Practically, we generate a random variable *u* with a given *ν* and examined different levels of scale *s* to match the median absolute error med|*e*| and the proportion of errors under 20% *#*(|*e*| < 0.2) observed in the Zillow data. We show the results in Table 2. The estimated degrees-of-freedom $\tilde \nu $ vary from a low of 1.06 (Philadelphia) to a high of 2.2 (Denver). For the Student *t*, the first moment (mean) exists when the degrees-of-freedom exceed 1 and the variance exists when the degrees-of-freedom exceed 2. All the cities have a mean that exists, but only three cities have a finite variance (Denver, Portland, and Seattle). None of the cities have a defined skewness or kurtosis. Further extending the results, we calculate that Pittsburgh has 5.2% of the predictions that exceed 50% error while Seattle has only 1.1% of the predictions that exceed 50% error.

Various studies have examined non-Gaussian error terms in house price models. Chasco et al. (2018) analyze the impact of different non-normal error distributions on a test for spatial groupwise heteroscedasticity through simulations. They also show a clear non-normality in the error terms using the Jarque-Bera statistic on houses prices in Madrid. As the distribution of house selling prices display heavy tails, De Oliveira and Ecker (2019) propose a non-Gaussian hedonic spatial model for the log selling prices of 1,502 houses in Cedar falls (Iowa). Aquaro et al. (2021) perform Monte Carlo simulations to analyse the impact of non-Gaussian errors in a spatial house price model with heterogeneous coefficients.

The lack of normality in the error terms has major implications for prediction intervals. For prediction, a key distinction exists between the confidence interval of the prediction and a prediction interval. A confidence interval for the prediction expresses the uncertainty associated with the prediction, mainly due to a low sample size. As the sample size increases $n \rightarrow \infty $, the prediction becomes less and less variable and, at some point, goes to a constant. If the model is correct, with enough data, the model will yield $\hat y=E(y)$ with no uncertainty. However, a prediction interval takes into account the uncertainly associated with the prediction and, even more importantly, the uncertainty associated with the disturbance. In the case of enough data, the distribution of *y*_*i*_ is just *E*(*y*_*i*_) plus the distribution of the disturbance which could be highly non-normal. In this case, increasing the sample size reduces the uncertainty of the forecast but does not have any effect on the uncertainty associated with the disturbance. Typical prediction intervals often use the standard deviation *σ* of the random variable *y*_*i*_ and follow the form $y_{i} =\hat y_{i} \pm g\cdot \hat \sigma $ where *g* captures the desired level of accuracy. However, the Student *t* distributed random variables do not have a defined variance for less than two degrees-of-freedom, and so this would not work for the Zillow data. Of course, one can always define a prediction interval using the quantiles of a non-normal distribution, and it will assign substantially more probability to extreme values than with the normal distribution Table 1.

**Table 1:** Estimated Student *t* for Zillow Errors in 2016

	$\tilde \nu $	$\tilde s$	av(|*e*| > 0.5)	Med |*e*|	av(|*e*| < 0.2)
National	1.3711	0.0503	0.0303	0.0450	0.8970
Atlanta	1.1987	0.0440	0.0362	0.0410	0.8940
Baltimore	1.2642	0.0369	0.0254	0.0340	0.9200
Boston	1.6032	0.0524	0.0211	0.0450	0.9130
Charlotte	1.5734	0.0590	0.0267	0.0510	0.8930
Chicago	1.2096	0.0505	0.0416	0.0470	0.8770
Cincinnati	1.1923	0.0503	0.0430	0.0470	0.8740
Cleveland	1.2175	0.0387	0.0297	0.0360	0.9100
DFW	1.6928	0.0401	0.0115	0.0340	0.9470
Denver	2.1893	0.0623	0.0116	0.0500	0.9250
Detroit	1.3656	0.0491	0.0297	0.0440	0.8990
Miami	1.7752	0.0680	0.0247	0.0570	0.8860
Minneapolis	1.8725	0.0495	0.0120	0.0410	0.9370
New York	1.3073	0.0550	0.0385	0.0500	0.8760
Orlando	1.4991	0.0434	0.0191	0.0380	0.9260
Philadelphia	1.0605	0.0428	0.0475	0.0420	0.8750
Phoenix	1.5218	0.0357	0.0136	0.0310	0.9460
Pittsburgh	1.0776	0.0482	0.0518	0.0470	0.8620
Portland	2.1256	0.0496	0.0079	0.0400	0.9490
Riverside	1.2738	0.0425	0.0296	0.0390	0.9060
Sacramento	1.6066	0.0536	0.0217	0.0460	0.9100
San Diego	1.4828	0.0571	0.0296	0.0500	0.8900
San Francisco	1.8053	0.0599	0.0189	0.0500	0.9090
Seattle	2.0109	0.0540	0.0112	0.0440	0.9350
Tampa	1.3153	0.0506	0.0340	0.0460	0.8890
D.C.	1.7042	0.0413	0.0119	0.0350	0.9450
Los Angeles	1.4670	0.0443	0.0211	0.0390	0.9210

The time span for the observations that Zillow reported on September 30, 2016 was not clear. To see whether this was sensitive to the unknown time span we estimated the statistics again using a new sample from data publicly provided by Zillow and last updated on June 26, 2019. The results here differ somewhat from those reported in Table 2 as we only focus on off-market properties that are more difficult to evaluate as documented by the error statistics. However, the results in Table 2 still show substantial kurtosis with the maximum degrees-of-freedom for Seattle at 2.22 (versus 2.19 for Denver in the 2016 data) and the minimum degrees-of-freedom of 1.37 for Baltimore (versus Philadelphia at 1.06 in the 2016 data).

**Table 2:** Estimated Student *t* for Zillow Errors in 2019

	D.F.	Scale	av(|*e*| > 0.5)	Med |*e*|	av(|*e*| < 0.2)
Atlanta	1.4133	0.0811	0.0544	0.0720	0.8170
Baltimore	1.3674	0.0748	0.0523	0.0670	0.8290
Boston	2.1096	0.0915	0.0284	0.0740	0.8460
Charlotte	1.4165	0.0756	0.0492	0.0670	0.8330
Chicago	1.8832	0.0979	0.0410	0.0810	0.8140
Cincinnati	1.5955	0.1015	0.0600	0.0870	0.7810
Cleveland	2.0133	0.0282	0.0031	0.0230	0.9810
Denver	2.1956	0.0685	0.0140	0.0550	0.9100
Detroit	1.6072	0.1025	0.0599	0.0880	0.7790
Los Angeles	1.6662	0.0718	0.0315	0.0610	0.8680
Miami	1.5942	0.0850	0.0456	0.0730	0.8260
Minneapolis	1.6900	0.0755	0.0332	0.0640	0.8600
New York	1.6607	0.1118	0.0648	0.0950	0.7600
Orlando	1.6302	0.0689	0.0312	0.0590	0.8720
Philadelphia	1.3926	0.0974	0.0717	0.0870	0.7700
Phoenix	1.7671	0.0703	0.0265	0.0590	0.8800
Pittsburgh	1.3717	0.1341	0.1122	0.1200	0.6750
Portland	1.7336	0.0688	0.0267	0.0580	0.8810
Riverside	1.4154	0.0619	0.0374	0.0550	0.8700
Sacramento	1.5618	0.0670	0.0331	0.0580	0.8710
San Diego	1.7491	0.0666	0.0247	0.0560	0.8880
San Francisco	1.9333	0.0876	0.0317	0.0720	0.8460
Seattle	2.2244	0.0799	0.0189	0.0640	0.8830
Tampa	1.6940	0.0955	0.0486	0.0810	0.8060
Washington	1.7545	0.0585	0.0197	0.0490	0.9090

## The Kurtosis in Regression Models with Spatial and Temporal Dependence

In this section we examine the kurtosis in regression models with spatial and temporal dependence. “The Kurtosis of Linear Combinations” provides a theorem to compute the kurtosis for linear combinations that will be used for spatial and temporal autoregressive and moving average processes and examines the attenuation of kurtosis that arises from models that ignore temporal, spatial, and spatiotemporal dependence. “Monte Carlo study” shows these points with specific distributions and dependence data generating processes. “The relationship between goodness-of-fit and kurtosis” describes the relationship between the goodness-of-fit of a regression model and the kurtosis of the disturbances. We also show how introducing many explanatory variables in a regression model may have the effect of raising the level of non-normality in the residuals.

### The Kurtosis of Linear Combinations

The following theorem is helpful to analyse the kurtosis of a spatial and temporal autoregressive and moving average processes.

#### **Theorem 3.1**

Let ***r*** be a vector of *k* unit symmetric and mutually independent random variables (rvs) with level of kurtosis ***κ***. We consider a linear combination of ***r***

3.1 $$ v=\boldsymbol{r}^{\prime}\boldsymbol{p}  $$

where the vector $\boldsymbol {p}=\begin {bmatrix}p_{1}&p_{2}&{\ldots } &p_{k}\end {bmatrix}^{\prime } $ represents the weights of the linear combination Eq. 3.1. The excess kurtosis of *v* is

3.2 $$ \kappa_{v}-3=\frac{(\boldsymbol{\kappa}-3)' \boldsymbol{p}^{(4)} }{{\sigma^{4}_{v}}} $$

where

- $\boldsymbol {\kappa }=\begin {bmatrix}\kappa _{1}&\kappa _{2}&{\ldots } &\kappa _{k}\end {bmatrix}^{\prime }$ contains the kurtosis of the rvs *r*_*j*_ with *j* = 1,2,...,*k*
- $\boldsymbol {p}^{(q)}=\begin {bmatrix}{p^{q}_{1}}&{p^{q}_{2}}&{\ldots } &{p^{q}_{k}}\end {bmatrix}^{\prime }$ contains the *q* th powers of the weights *p*_*j*_ with *j* = 1,2,...,*k*
- ${\sigma ^{4}_{v}}=[{\sigma ^{2}_{v}}]^{2}$ is the square of the variance of *v*.

#### *Proof*

The moments of the rvs *r*_*j*_ with *j* = 1,2,...,*k* are

3.3 $$ \begin{array}{@{}rcl@{}} E({r^{q}_{j}})&=0, \text{ for } q=1,3, \ j=1, \ldots, k \end{array} $$

3.4 $$ \begin{array}{@{}rcl@{}} E({r^{q}_{j}})&=1, \text{ for } q=2, \ j=1, \ldots, k \end{array} $$

3.5 $$ \begin{array}{@{}rcl@{}} E({r^{q}_{j}})&=\kappa_{j}, \text{ for } q=4, \ j=1, \ldots, k. \end{array} $$

Given that ***r*** contains mutually independent unit rvs *r*_*j*_, the variance of *v* equals the sum of squared weights

3.6 $$ \begin{array}{@{}rcl@{}} {\sigma^{2}_{v}}&=\boldsymbol{\iota}_{k}^{\prime}\boldsymbol{p}^{(2)} \end{array} $$

where ***ι***_*k*_ is a vector composed of ones and ***p***^(2)^ contains the squares of the weights *p*_*j*_ with *j* = 1,2,...,*k*.

The kurtosis of *v* defined in Eq. 3.1 is

3.7 $$ \kappa_{v}=\frac{E(v^{4})}{{\sigma^{4}_{v}}}     \text{where}   v^{4}=(p_{1}r_{1}+p_{2}r_{2}+\ldots+p_{k}r_{k})^{4}  $$

We can compute the fourth power of *v* in Eq. 3.7 using the multinomial theorem as stated in Eq. 3.8.

3.8 $$ (p_{1}r_{1}+p_{2}r_{2}+\ldots+p_{k}r_{k})^{4}= \sum\limits_{s_{1}+s_{2}+\ldots+s_{k}=4}\binom{4}{s_{1}, s_{2}, \dots,s_{k}}\prod\limits_{t=1}^{k} (p_{t}r_{t})^{s_{t}}  $$

Because the odd moments equal 0, as Eq. 3.3 shows, *E*(*v*^4^) involves only even powers. For the fourth power, this does not involve cross-products and therefore the contribution to *E*(*v*^4^) from the fourth powers equals $\boldsymbol {\kappa }^{\prime }\boldsymbol {p}^{(4)}$. The only other non-zero terms are associated with the products of $\boldsymbol {p}_{i}^{(2)}\boldsymbol {p}_{j}^{(2)}$ since $E({r_{i}^{2}}{r_{j}^{2}})=1$ with *i*,*j* = 1,2,…*k*. Thus, there will be 4!/(2!2!) = 6 quadratic terms. In summary, there will be 0 first order terms, 6 quadratic terms, 0 cubic terms, and 1 quartic term in the overall polynomial in Eq. 3.8.

In actually computing the polynomial, we can easily do this via some linear algebra. We begin by defining the matrix *P* in Eq. 3.9 as the outer product of the squared weights $\boldsymbol {p}^{2}_{j}$,

3.9 $$ \begin{array}{@{}rcl@{}} P&= \left( \boldsymbol{p}^{(2)}\right)\cdot \left( \boldsymbol{p}^{(2)}\right)' \end{array} $$

We also consider the *k* by *k* matrix *D* whose elements on the main diagonal are given by the fourth powers of the weights *p*_*j*_ with *j* = 1,2,⋯ ,*k* and all other elements are zeros

$$D={diag}(\boldsymbol{p}^{(4)}).$$

The upper and lower triangles of the matrix *P* contains all the cross-products of $\boldsymbol {p}_{i}^{(2)}\boldsymbol {p}_{j}^{(2)}$ for *i*,*j* = 1,2…*k*. The matrix (*P* − *D*) eliminates the fourth order products on the diagonal, and therefore $\boldsymbol {\iota }_{k}^{\prime }(P-D)\boldsymbol {\iota }_{k}$ contains 2 times $\boldsymbol {p}_{i}^{(2)}\boldsymbol {p}_{j}^{(2)}$ for *i*,*j* = 1,2,…*k*. From the multinomial coefficient in Eq. 3.8, the multiplicity of the products of the quadratic terms $\boldsymbol {p}_{i}^{(2)}\boldsymbol {p}_{j}^{(2)}$ in a fourth order expansion equal 4!/(2!2!) = 6. Hence, the quantity $3 \boldsymbol {\iota }_{k}^{\prime }(P-D)\boldsymbol {\iota }_{k}$ contains all the second order cross-products $\boldsymbol {p}_{i}^{(2)}\boldsymbol {p}_{j}^{(2)}$ with the desired multiplicity. Therefore, this allows to simply the expression of *E*(*v*^4^) as follows

3.10 $$ \begin{array}{@{}rcl@{}} E(v^{4})&=\boldsymbol{\kappa}^{\prime} \boldsymbol{p}^{(4)} + 3 \boldsymbol{\iota}_{k}^{\prime}(P-D)\boldsymbol{\iota}_{k} \end{array} $$

Given the definition of kurtosis in Eq. 3.7 and the expression of *E*(*v*^4^) from Eq. 3.10 as well as ${\sigma ^{2}_{v}}$ from Eq. 3.6, this leads to the kurtosis of *v* in Eq. 3.11.

3.11 $$ \begin{array}{@{}rcl@{}} \kappa_{v}&=\frac{\boldsymbol{\kappa}^{\prime} \boldsymbol{p}^{(4)} + 3 \boldsymbol{\iota}_{k}^{\prime}(P-D)\boldsymbol{\iota}_{k}}{{\sigma^{4}_{v}}} \end{array} $$

Some manipulations of Eq. 3.11 leads to a simpler expression in Eq. 3.12.

3.12 $$ \begin{array}{@{}rcl@{}} \kappa_{v}&=&\frac{\boldsymbol{\kappa}^{\prime} \boldsymbol{p}^{(4)} + 3 \boldsymbol{\iota_{k}}^{\prime}P\boldsymbol{\iota_{k}}-3\boldsymbol{\iota^{\prime}_{k}}D\boldsymbol{\iota_{k}}}{{\sigma^{4}_{v}}} \\ &=&\frac{\boldsymbol{\kappa}^{\prime} \boldsymbol{p}^{(4)} + 3 \boldsymbol{\iota_{k}}^{\prime}\left( \boldsymbol{p}^{(2)}\right)\cdot \left( \boldsymbol{p}^{(2)}\right)'\boldsymbol{\iota_{k}}-3\boldsymbol{\iota^{\prime}_{k}}\boldsymbol{p}^{(4)}}{{\sigma^{4}_{v}}} \\ &=&\frac{(\boldsymbol{\kappa} -3\boldsymbol{\iota_{k}})'\boldsymbol{p}^{(4)} + 3 \boldsymbol{\iota_{k}}^{\prime}\left( \boldsymbol{p}^{(2)}\right)\cdot \left( \boldsymbol{p}^{(2)}\right)'\boldsymbol{\iota_{k}}}{{\sigma^{4}_{v}}} \\ &=&\frac{(\boldsymbol{\kappa}-3)' \boldsymbol{p}^{(4)} + 3 {\sigma^{4}_{v}}}{{\sigma^{4}_{v}}} \\ &=&\frac{(\boldsymbol{\kappa}-3)' \boldsymbol{p}^{(4)} }{{\sigma^{4}_{v}}}+3 \\ \kappa_{v}-3&=&\frac{(\boldsymbol{\kappa}-3)' \boldsymbol{p}^{(4)} }{{\sigma^{4}_{v}}} \end{array} $$

where the excess kurtosis of *v* is a function of the weighted excess kurtosis of the component random variables ***r***. □

We can obtain a further simplification by examining a combination of random variables *v*_*eq*_ where each random variable *r*_*j*_ with *j* = 1,2,…,*k* shows the same level of kurtosis *κ*_*j*_ = *κ* and each random variable has equal weights *p*_*j*_ = 1/*k* with *j* = 1,2,…,*k*. In other words, let $v_{eq}={\sum }_{j=1}^{k} r_{j}/k$. In this case, the kurtosis approaches 3 as the number of random variables *k* becomes large as shown in Eq. 3.13

3.13 $$ \kappa_{eq}=3+\frac{\kappa-3}{k}. $$

We now turn to examining cases where each random variable has the same level of kurtosis *κ*, but the linear combination has different weights so that $v=\boldsymbol {\kappa }^{\prime }\boldsymbol {p}=\kappa \boldsymbol {\iota _{k}}^{\prime }\boldsymbol {p}$. Again, in the spirit of seeking simple expressions for kurtosis, we examine the ratio *ϕ* between the kurtosis of the weighted random variable $v=\boldsymbol {r}^{\prime }\boldsymbol {p}$ relative to the excess kurtosis *κ* − 3 for each random variable. From Eq. 3.2 we obtain Eqs. 3.14 and 3.15

3.14 $$ \begin{array}{@{}rcl@{}} \phi&=&\frac{(\kappa-3)^{-1}(\boldsymbol{\kappa}-3)' \boldsymbol{p}^{(4)} }{{\sigma^{4}_{v}}}=\frac{(\kappa-3)^{-1}(\kappa-3)\boldsymbol{\iota_{k}}^{\prime} \boldsymbol{p}^{(4)} }{{\sigma^{4}_{v}}} \end{array} $$

3.15 $$ \begin{array}{@{}rcl@{}} &=&\frac{\boldsymbol{\iota_{k}}^{\prime} \boldsymbol{p}^{(4)} }{\left( \boldsymbol{\iota}_{k}^{\prime}\boldsymbol{p}^{(2)}\right)^{2}}. \end{array} $$

To make this more concrete, we illustrate the relative levels of kurtosis *ϕ* for two temporal processes, a Moving Average MA(1) and an Autoregressive AR(1). For a temporal MA(1) process, the weight vector is $\boldsymbol {p}=\left [\begin {array}{ll}1 & \tau \end {array}\right ]^{\prime }$, the variance of *v* is ${\sigma ^{2}_{v}}=1+\tau ^{2}$ and the sum of the fourth moments is $\boldsymbol {\iota }_{k}^{\prime }\boldsymbol {p}^{(4)}=1 + \tau ^{4}$. We assume *τ* ∈ (− 1,1). We obtain an expression for the relative excess kurtosis in Eq. 3.16

3.16 $$ \phi_{MA(1),t}=\frac{\kappa_{v}-3}{\kappa-3}=\frac{1+\tau^{4}}{(1+\tau^{2})^{2}}. $$

For any level of *τ*, *ϕ*_*MA*(1),*t*_ ≤ 1 and, therefore, the level of kurtosis in the random variable *v* decreases from independent *r* to a temporal moving average dependence process. For example, *τ* = 0.5 leads to a *ϕ*_*MA*(1),*t*_ = 0.68.

If we consider a temporal MA(2) process, the weight vector becomes $\boldsymbol {p}=\left [\begin {array}{lll}1 & \tau _{1} & \tau _{2} \end {array}\right ]^{\prime }$. The variance of *v* is ${\sigma ^{2}_{v}}=1+{\tau _{1}^{2}}+{\tau _{2}^{2}}$ and the sum of the fourth moments is $\boldsymbol {\iota }_{k}^{\prime }\boldsymbol {p}^{(4)}=1 + {\tau _{1}^{4}}+{\tau _{2}^{4}}$. If the MA(2) process is invertible, the relative excess kurtosis in Eq. 3.15 is

3.17 $$ \phi_{MA(2),t}=\frac{\kappa_{v}-3}{\kappa-3}=\frac{1+{\tau_{1}^{4}}+{\tau_{2}^{4}}}{(1+{\tau^{2}_{1}}+{\tau^{2}_{2}})^{2}}. $$

If we consider a MA(q) process that is invertible, the Eq. 3.17 can be generalised for a temporal MA(q) process as in Eq. 3.18

3.18 $$ \phi_{MA(q),t}=\frac{\kappa_{v}-3}{\kappa-3}=\frac{1+{\tau_{1}^{4}}+{\tau_{2}^{4}}+...+{\tau_{q}^{4}}}{(1+{\tau^{2}_{1}}+{\tau^{2}_{2}}+...+{\tau_{q}^{2}})^{2}}. $$

For a temporal AR(1) process, the weight vector is $\boldsymbol {p}=\left [ 1 \tau \tau ^{2} \tau ^{3} {\ldots } \right ]^{\prime }$ while $\boldsymbol {p^{(4)}}=\left [ 1 \tau ^{4} \tau ^{8} \tau ^{12} {\ldots } \right ]^{\prime }$. Therefore, the sum of the fourth moments is $\boldsymbol {\iota }_{k}^{\prime }\boldsymbol {p}^{(4)}=(1-\tau ^{4})^{-1}$. Turning to the variance, $\boldsymbol {p^{(2)}}=\left [ 1 \tau ^{2} \tau ^{4} \tau ^{6} {\ldots } \right ]^{\prime }$ which means ${\sigma ^{2}_{v}}=\boldsymbol {\iota }_{k}^{\prime }\boldsymbol {p}^{(2)}=(1-\tau ^{2})^{-1}$. Therefore, we obtain a simple expression for the relative excess kurtosis in Eq. 3.19

3.19 $$ \phi_{AR(1),t}=\frac{(1-\tau^{4})^{-1}}{(1-\tau^{2})^{-2}}=\frac{1-2\tau^{2}+\tau^{4}}{1-\tau^{4}}. $$

For *τ* ∈ (− 1,1), *ϕ*_*AR*(1),*t*_ < 1 which means the autoregressive process reduces the level of kurtosis in the random variable *v* relative to an independent *r*. For example, *τ* = 0.5 leads to a *ϕ*_*AR*,*t*_ = 0.6.

The relative excess kurtosis in Eq. 3.15 for a AR(p) process is

$$ \phi_{AR(p),t}=\frac{1-{\tau^{2}_{1}}-{\tau^{2}_{2}}+...-{\tau^{2}_{p}}}{1-{\tau_{1}^{4}}-{\tau_{2}^{4}}+...-{\tau_{p}^{4}}}. $$

Analogously, we consider spatial MA(1) and AR(1) processes. To define them, we assume an exogenous square spatial weight matrix *W* and the associated scalar parameter *ρ*. To keep this simple, we assume *I*_*n*_ − *ρW* is invertible for all *ρ* in the open interval (− 1,1). This condition is sufficient for row-stochastic *W* (which has a principal eigenvalue of 1) and symmetric *W* scaled to have a principal eigenvalue of 1. In the spatial weight matrix *W* the generic element *w*_*ij*_ is equal to a positive number when observation *j* is a neighbor to observation *i* and 0 otherwise. To prevent each observation from predicting itself, *w*_*ii*_ = 0 for all *i*.^3^ For simplicity, we assume that each row of *W* has a *m* entries of 1/*m* and zeros otherwise (*m* nearest neighbors). The disturbance vector for a spatial MA(1) process is given by ***u*** = (*I*_*n*_ + *ρW*)***ε*** where ***ε*** represents a vector of *iid* rvs with kurtosis *κ*. In this case the vector of weights ***p*** is $\boldsymbol {p}=\left [1 {\ldots } \rho /m {\ldots } \rho /m {\ldots } \right ]^{\prime }$, the variance of *v* is ${\sigma ^{2}_{v}}=1+{\sum }_{i=1}^{m}(p/ m)^{2}=1 + m^{-1}\rho ^{2}$ and the sum of the fourth moments is $\boldsymbol {\iota }_{k}^{\prime }\boldsymbol {p}^{(4)}=1+{\sum }_{i=1}^{m}(p/m)^{4}=1 + m^{-3}\rho ^{4}$. Hence, we obtain

3.20 $$ \phi_{MA(1),s}=\frac{1+m^{-3}\rho^{4}}{(1+m^{-1}\rho^{2})^{2}} \ . $$

For first-order spatial autoregressive model, the residual is given by ***u*** = (*I*_*n*_ − *ρW*)^− 1^***ε*** where ***ε*** represents a vector of *iid* rvs with kurtosis *κ*. To obtain a single row of (*I*_*n*_ − *ρW*)^− 1^, we need to solve the following equation $(I_{n}-\rho W^{\prime })\boldsymbol {x}=\boldsymbol {0}_{\slash i}$ for ***x*** where ***0***_/*i*_ is a vector of zeros but with a one on the *i* th row.^4^ After computing ***x***, its transpose $x^{\prime }$ represents the *i* th row of (*I*_*n*_ − *ρW*)^− 1^. Given this row, we can easily compute the variance of *v*, the sum of the fourth moments $\boldsymbol {\iota }_{k}^{\prime }\boldsymbol {p}^{(4)}$ and *ϕ*_*AR*(1),*s*_.

Analogously to the temporal processes, we can extend the results in Eq. 3.20 and for the *AR*(1) process to *MA*(*q*) and *AR*(*q*) spatial processes.

### Monte Carlo study

We perform a Monte Carlo study to better understand the reduction of excess kurtosis for a spatiotemporal process. We use a contiguity *W* matrix with 100 observations and generate a product separable spatialtemporal process where *L* represents a *n* × *n* matrix that lags a *n* × 1 vector *v*_*t*_ so that *Lv*_*t*_ = *v*_*t*− 1_,

$$ \boldsymbol{u}=\left( (I_{n}-\tau L)(I_{n}-\rho W)\right)^{-1}\boldsymbol{\varepsilon} $$

where ***ε*** is a vector of *iid* random variables. Because the time series expansion uses 75 terms and the spatial part uses 100 terms, each trial involves 7,500 ***ε*** vectors. We repeat this for 1,000 iterations and compute the empirical excess kurtosis $\hat \kappa _{v}-3$ and $\hat \phi $ defined in Eq. 3.14. We also derive a correction $\hat \phi _{\text {cor}}$ obtained by dividing $\hat \phi $ by the value of the same parameter $\hat \phi $ in the case of temporal and spatial independence (i.e. *ρ*,*τ* = 0). We report the *t*-stats for the difference between $\hat \phi _{\text {cor}}$ and *ϕ* in the following tables.

Table 3 displays the results for Laplace distributed disturbances with *κ* = 6, Table 4 contains the results for logistic disturbances with *κ* = 4.2, and Table 5 shows the results for Pearson disturbances with *κ* = 4. By and large, the empirical measurements of $\hat \phi $ agree with the theoretical calculations. The Laplace distribution shows close agreement between the measured and theoretical values of *ϕ*, with none of the *t* statistics showing significant differences.

**Table 3:** The Kurtosis for Autoregressive Spatiotemporal models with 6 nearest neighbors and Laplace disturbances

Case	*ρ*	*τ*	$\hat \kappa _{v}-3$	*ϕ*	$\hat \phi $	$\hat \phi _{\text {cor}}$	*t*_*ϕ*_
1	0.0	0.0	2.9920	1.0000	0.9973	1.0000	0.0000
2	0.0	0.4	2.1666	0.7241	0.7222	0.7241	− 0.0741
3	0.0	0.8	0.6567	0.2195	0.2189	0.2195	− 0.2500
4	0.4	0.0	2.7364	0.9146	0.9121	0.9146	− 0.1089
5	0.4	0.4	1.9815	0.6623	0.6605	0.6623	− 0.1693
6	0.4	0.8	0.6006	0.2008	0.2002	0.2007	− 0.5322
7	0.8	0.0	1.4499	0.4846	0.4833	0.4846	− 0.0687
8	0.8	0.4	1.0498	0.3509	0.3499	0.3509	− 0.3402
9	0.8	0.8	0.3180	0.1064	0.1060	0.1063	− 1.7036

**Table 4:** The Kurtosis for Autoregressive Spatiotemporal models with 6 nearest neighbors and Logistic disturbances

	*ρ*	*τ*	$\hat \kappa _{v}-3$	*ϕ*	$\hat \phi $	$\hat \phi _{\text {cor}}$	*t*_*ϕ*_
1	0.0000	0.0000	1.1970	1.0000	0.9975	1.0000	0.0000
2	0.0000	0.4000	0.8668	0.7241	0.7223	0.7241	− 0.3354
3	0.0000	0.8000	0.2624	0.2195	0.2187	0.2192	− 3.8955
4	0.4000	0.0000	1.0949	0.9147	0.9124	0.9146	− 0.1298
5	0.4000	0.4000	0.7928	0.6623	0.6606	0.6623	− 0.5227
6	0.4000	0.8000	0.2399	0.2008	0.1999	0.2004	− 4.9980
7	0.8000	0.0000	0.5834	0.4877	0.4862	0.4874	− 2.3209
8	0.8000	0.4000	0.4223	0.3531	0.3520	0.3528	− 2.9672
9	0.8000	0.8000	0.1275	0.1070	0.1063	0.1065	− 7.3347

**Table 5:** The Kurtosis for Autoregressive Spatiotemporal models with 6 nearest neighbors and Pearson disturbances

	*ρ*	*τ*	$\hat \kappa _{v}-3$	*ϕ*	$\hat \phi $	$\hat \phi _{\text {cor}}$	*t*_*ϕ*_
1	0.0	0.0	2.9406	1.0000	0.9802	1.0000	0.0000
2	0.0	0.4	2.1345	0.7241	0.7115	0.7259	0.6983
3	0.0	0.8	0.6520	0.2195	0.2173	0.2217	3.4776
4	0.4	0.0	2.6928	0.9153	0.8976	0.9157	0.1465
5	0.4	0.4	1.9546	0.6628	0.6515	0.6647	0.8379
6	0.4	0.8	0.5970	0.2009	0.1990	0.2030	3.6005
7	0.8	0.0	1.4443	0.4892	0.4814	0.4912	1.0837
8	0.8	0.4	1.0479	0.3543	0.3493	0.3563	1.5774
9	0.8	0.8	0.3193	0.1074	0.1064	0.1086	3.5689

We now turn to examining other spatial structures, specifically those with 15 and 30 nearest neighbors. Both of these weight matrices *W* were symmetricized and then made doubly stochastic so that the row and column sums equal 1. This is common procedure in spatial econometrics (LeSage and Pace 2009, p. 88). Table 6 contains the 15 nearest neighbor results with Laplace disturbances. The empirical and theoretical results agree closely.

**Table 6:** The Kurtosis for Autoregressive Spatiotemporal models with 15 nearest neighbors and Laplace disturbances

	*ρ*	*τ*	$\hat \kappa _{v}-3$	*ϕ*	$\hat \phi $	$\hat \phi _{\text {cor}}$	*t*_*ϕ*_
1	0.0	0.0	2.9910	1.0000	0.9970	1.0000	0.0000
2	0.0	0.4	2.1656	0.7241	0.7219	0.7240	− 0.3159
3	0.0	0.8	0.6559	0.2195	0.2186	0.2193	− 1.8767
4	0.4	0.0	2.8787	0.9625	0.9596	0.9625	− 0.1802
5	0.4	0.4	2.0844	0.6970	0.6948	0.6969	− 0.4533
6	0.4	0.8	0.6313	0.2113	0.2104	0.2111	− 1.9137
7	0.8	0.0	1.7478	0.5846	0.5826	0.5844	− 0.9353
8	0.8	0.4	1.2654	0.4233	0.4218	0.4231	− 1.3037
9	0.8	0.8	0.3832	0.1283	0.1277	0.1281	− 2.2095

If we consider *τ* = *ρ* = 0.8, Table 3 shows a lower value of *ϕ* (0.1064) for 15 nearest neighbors compared to the value of *ϕ* (0.1281) for 30 nearest neighbors reported in Table 7. We interpret these results considering that the larger number of neighbors uses more random variables, but this also reduces the variance of the average which weakens the reduction of kurtosis.

**Table 7:** The Kurtosis for Autoregressive Spatiotemporal models with 30 nearest neighbors and Laplace disturbances

	*ρ*	*τ*	$\hat \kappa _{v}-3$	*ϕ*	$\hat \phi $	$\hat \phi _{\text {cor}}$	*t*_*ϕ*_
1	0.0	0.0	2.9894	1.0000	0.9965	1.0000	0.0000
2	0.0	0.4	2.1649	0.7241	0.7216	0.7242	0.0685
3	0.0	0.8	0.6563	0.2195	0.2188	0.2195	0.1070
4	0.4	0.0	2.9274	0.9793	0.9758	0.9792	− 0.0567
5	0.4	0.4	2.1202	0.7092	0.7067	0.7092	0.1074
6	0.4	0.8	0.6428	0.2150	0.2143	0.2150	0.1915
7	0.8	0.0	2.0543	0.6877	0.6848	0.6872	− 0.6327
8	0.8	0.4	1.4884	0.4980	0.4961	0.4979	− 0.1037
9	0.8	0.8	0.4519	0.1509	0.1506	0.1512	0.8087

### The relationship between goodness-of-fit and kurtosis

The purpose of this section is to show how goodness-of-fit can amplify (or attenuate) the kurtosis in the residuals of spatiotemporal processes. We consider $y=\boldsymbol {r}^{\prime }\boldsymbol {p}$ a linear combination of $\boldsymbol {r}=\left [\begin {array}{ll} \hat y & \hat e \end {array}\right ]^{\prime }$ with weights $\boldsymbol {p}=\left [\begin {array}{ll} \sigma _{\hat y}& \sigma _{\hat e} \end {array}\right ]^{\prime }$. We begin with a simple setting where *E*(*y*) = 0 with ${\sigma _{y}^{2}}=1$, so we obtain $\sigma ^{2}_{\hat y}=R^{2}$, $\sigma ^{2}_{\hat e}=1-R^{2}$ and $\boldsymbol {p}=\left [\begin {array}{ll} R & (1-R^{2})^{1/2} \end {array}\right ]^{\prime }$. Let $\hat y$ display a level of kurtosis equal to $\kappa _{\hat y}$ and $\hat e$ display a level of kurtosis equal to $\kappa _{\hat e}$.

Given the weights ***p*** and the levels of kurtosis $\kappa _{\hat y}$ and $\kappa _{\hat e}$, we substitute these values in the Eq. 3.2 and we obtain Eq. 3.21

3.21 $$ \kappa_{y}-3=R^{4}(\kappa_{\hat y}-3)+\left( 1-R^{2}\right)^{2}\left( \kappa_{\hat e}-3\right).  $$

If we solve for the kurtosis of the residuals $\kappa _{\hat e}$ in Eq. 3.21, we obtain Eqs. 3.22 and 3.23

3.22 $$ \begin{array}{@{}rcl@{}} \kappa_{\hat e}-3&=&\frac{(\kappa_{y}-3)-R^{4}(\kappa_{\hat y}-3)}{\left( 1-R^{2}\right)^{2}} \end{array} $$

3.23 $$ \begin{array}{@{}rcl@{}} &=&\frac{\kappa_{y}-\kappa_{\hat y}+\left( 1-R^{4}\right)(\kappa_{\hat y}-3)}{\left( 1-R^{2}\right)^{2}} \end{array} $$

We can give further structure to Eq. 3.23 by modeling the $\kappa _{\hat y}$. Intuitively, elaborate regression models may contain a large number of explanatory variables and the excess kurtosis of a linear combination of a large number of explanatory variables could go to 0. This is also the situation that often produces a high coefficient of determination *R*^2^. If $\hat y$ is normal distributed, therefore its kurtosis is 3 and the Eq. 3.23 reduces to Eqs. 3.24 and 3.25

3.24 $$ \begin{array}{@{}rcl@{}} \kappa_{\hat e}-3&=&\alpha \cdot (\kappa_{y}-3) \end{array} $$

3.25 $$ \begin{array}{@{}rcl@{}} \alpha&=&\frac{1}{\left( 1-R^{2}\right)^{2}}. \end{array} $$

Equation 3.25 shows that any excess kurtosis in *y* is amplified by *α* in terms of excess kurtosis in the residuals. For example, if *R*^2^ = 0.9, the residuals will excess kurtosis augmented by 100 times higher than the excess kurtosis of *y*. This declines rapidly with fit, so that a coefficient of determination *R*^2^ = 0.5 results in a factor of four amplification of the excess kurtosis of $\hat e$ relative to the excess kurtosis of *y*.

We can go further in modeling the $\kappa _{\hat y}$ introducing assumptions on the kurtosis of individual explanatory variables and on the regression parameters. To illustrate this, we assume that $\hat y$ uses a large number of independent explanatory variables *x*_*a*_ where *E*(*x*_*a*_) = *E*(*y*) = 0, *x*_*a*_ ⊥ *x*_*b*_ for *a*,*b* = 1,…*k*. We can think of this as coming out of a principal components analysis where each regressor is orthogonal to the others. Furthermore, we can assume that the magnitude of the estimated regression coefficients follows a geometric decline where the most important component has a parameter of 1, the second most important component has a contribution of *g*, and so forth. These assumptions are represented by Eq. 3.26

3.26 $$ \hat y=x_{1}+x_{2}\cdot g+x_{3}\cdot g^{2}+x_{4} \cdot g^{3}+ \ldots, \ 0 \le g<1, $$

where *g* is a scalar constant giving the rate of the geometric decline in the contribution of *x*_*i*_. If the number of explanatory variables *x*_*i*_ is high enough, the sum in Eq. 3.26 converges. We also assume a constant level of kurtosis and variance for each *x*_*i*_ as well as that all the odd moments equal 0.

To compute the excess kurtosis of $\hat y$ given in Eq. 3.27, we can employ the same development of the convergence of an autoregressive sequence AR(1) shown in Eq. 3.20. Even if in this case there is no temporal dependence, we can use the same geometrically declining weights that occurs in an AR(1) process to obtain the following result

3.27 $$ \kappa_{\hat y}-3 = \frac{\left( 1-g^{2}\right)^{2}}{ 1-g^{4} }\cdot \left( \kappa_{x}-3\right ). $$

The slower the weights decline, the less excess kurtosis will be shown by $\hat y$. Equation 3.27 also shows that if the weight *g* = 0, the excess kurtosis of the prediction $\kappa _{\hat y}-3$ equal the excess kurtosis of the explanatory variable *κ*_*x*_ − 3. On the contrary, for a weight *g* close to 1, the excess kurtosis of the prediction will be close to 0, just as with a normal random variable. From the excess kurtosis of the residuals in Eq. 3.22 and the excess kurtosis of the prediction $\kappa _{\hat y}$, we derive the Eq. 3.28

3.28 $$ \kappa_{\hat e}-3=\alpha \cdot \left ((\kappa_{y}-3) - R^{4}\cdot\frac{\left( 1-g^{2}\right)^{2}}{ 1-g^{4} } \cdot \left( \kappa_{x}-3 \right) \right ) $$

As a specific example, we assume *g* = 0.9, *R*^2^ = 0.9, *κ*_*x*_ = 6, and *κ*_*y*_ = 4. These parameters would lead to $\kappa _{\hat y}=3.315$ and $\kappa _{\hat e}=82.3$. Changing *g* = 0.8 and *R*^2^ to 0.8 would yield $\kappa _{\hat e}=41.5$.

The Eq. 3.28 provides an explanation for why high fit regressions can have leptokurtic residuals. First, high fit (high *R*^2^) directly amplifies any excess kurtosis in *y* by large factors as *R*^2^ approaches 1. Second, if the high fit comes from including many explanatory variables, the excess kurtosis of the explanatory variables, which can reduce the excess kurtosis of the residuals, may tend to go away. In that sense, adding an additional explanatory variable increases *R*^2^ as well as decreases $\kappa _{\hat y}$ and, thus, increases the kurtosis of the residuals.

To illustrate this further, we conduct a small Monte Carlo experiment where *g* = 0.75,0.85,0.95, *R*^2^ = 0.5,0.75,0.95 and each entry represents the average of 25,000 trials with *n* = 100,000. We consider 100 explanatory variable *x*_*i*_ with different weights given by the powers of *g*. The results appear in Table 8. The third column *κ*_*e*_ in Table 8 represents the actual average kurtosis of the error term, generated using Laplace random variables with a theoretical kurtosis of 6. The fourth column represents its theoretical prediction according to the values of $\hat y$ and *y*, the fifth column contains the empirical value for the kurtosis of the predictions while the sixth column contains the predicted kurtosis of the predictions. Finally, the seventh column reports the measured kurtosis of *y* while the last column contains the predicted kurtosis of *y*. The empirical and theoretical values are in close agreement with each other and there are no statistically significant violations based on *t* tests on the differences. From Table 8 we note that a small amount of excess kurtosis in *y* with an almost identical amount of excess kurtosis in the predictions can lead to a kurtosis of 6 in the disturbances.

**Table 8:** Kurtosis Regression Statistics

Case	*g*	*R*^2^	*κ*_*e*_	$\hat \kappa _{\hat e}$	$\kappa _{\hat y}$	$\hat \kappa _{\hat y}$	*κ*_*y*_	$\hat \kappa _{y}$
1	0.7500	0.5000	5.9992	5.9986	3.8402	3.8400	3.9597	3.9598
2	0.7500	0.7500	5.9999	5.9962	3.8400	3.8400	3.6598	3.6600
3	0.7500	0.9500	6.0001	5.9886	3.8399	3.8400	3.7656	3.7656
4	0.8500	0.5000	6.0009	6.0003	3.4831	3.4833	3.8709	3.8710
5	0.8500	0.7500	5.9982	5.9929	3.4827	3.4833	3.4589	3.4592
6	0.8500	0.9500	5.9990	5.9492	3.4831	3.4833	3.4436	3.4437
7	0.9500	0.5000	6.0001	5.9996	3.1535	3.1537	3.7883	3.7885
8	0.9500	0.7500	5.9991	5.9969	3.1534	3.1537	3.2738	3.2739
9	0.9500	0.9500	5.9992	6.0379	3.1538	3.1537	3.1463	3.1463

## An Empirical Example

In this section we provide an empirical example of the positive relation between better fitting models and leptokurtic residuals. For example, the residuals of a model with only the intercept include the true disturbances as well as the omitted variables. Both the true disturbances and omitted variables may exhibit spatial, temporal, and spatiotemporal dependence across observations. The combination of these various disturbances can produce residuals that follow a more normal distribution than the underlying innovations and thus mask the underlying innovations. Consequently, modeling these aspects of the data can uncover or unmask the distribution, normal or non-normal, of the underlying innovations.

To show this, we use 62,266 census-tract level observations from the 2000 Census. The same sample has been used by LeSage and Pace (2009, p. 272). We consider a regression model where the dependent variable *y*_2000_ is the logarithm of the median housing price for census tracts in 2000. The regressor set *X*_1990_ is given by the logarithm of the following variables evaluated in 1990: house age, employment, median years of education, median age of the population, total households, white households, median household income, population, owner occupied housing units, renter occupied housing units, and census tract area. We show percentiles (1 through 99) of the variables in Table 9.

**Table 9:** Percentiles of logged explanatory variables

	1	10	25	50	75	90	99
*P*_2*K*_	10.292	10.882	11.212	11.573	11.995	12.400	13.321
*P*_90_	9.770	10.431	10.758	11.170	11.713	12.183	12.982
House Age	1.386	2.639	2.639	3.178	3.526	4.078	4.078
Employment	5.441	66659	7.068	7.437	7.757	8.012	8.361
Years Education	2.398	2.485	2.485	2.485	2.565	2.565	2.833
Population Age	3.091	3.296	3.466	3.466	3.676	3.676	3.902
HouseHolds	5.308	66524	66893	7.220	7.504	7.735	8.087
White HHs	3.296	66129	66829	7.282	7.627	7.896	8.275
HH Income	9.039	9.712	9.987	10.275	10.561	10.818	11.278
Population	7.102	8.306	8.681	9.013	9.297	9.529	9.875
Owner Occupied	4.007	5.790	66365	66796	7.124	7.373	7.717
Renter Occupied	3.219	4.718	5.328	5.935	66491	66935	7.659
Area of Tract	12.010	13.693	14.465	15.504	17.696	19.377	21.418

Given the dependent and explanatory variables, we examined different specifications to obtain some variation in goodness-of-fit as measured by $\tilde \sigma $ or median absolute residuals to examine whether this led to a corresponding variation in the distribution of the residuals as measured by the skewness, kurtosis, and the degrees-of-freedom from a fitted *t* distribution. Panel A in Table 10 shows the 12 different estimated specifications which involve various combinations of *X*, *W* *X*, temporal lags, spatial lags, and a spatial autoregressive error term as selected by the respective indicators as shown in Eq. 4.14.1

**Table 10:** Estimated Non-normality by Specification

Panel A: Specification					
Case	*k*				
1	1	0	0	0	0
2	2	1	0	0	0
3	2	0	0	1	0
4	3	1	0	1	0
5	12	0	1	0	0
6	13	1	1	0	0
7	13	0	1	1	0
8	14	1	1	1	0
9	23	0	1	0	1
10	24	1	1	0	1
11	25	0	1	1	1
12	26	1	1	1	1
Panel B: Statistical Performance					
Case	$\tilde \sigma $	Kurt	Skew	Med|*e*|	$\tilde \nu $
1	0.6138	3.5522	0.3830	0.3921	12.3744
2	0.2589	8.1560	0.0962	0.1357	3.9402
3	0.2810	18.6137	1.4036	0.1563	4.5736
4	0.2041	30.8543	1.4414	0.0870	2.6419
5	0.3873	7.5701	0.6404	0.2225	5.1410
6	0.2161	12.0014	0.0485	0.1074	3.3901
7	0.2675	17.6475	1.1653	0.1456	4.3290
8	0.1922	27.7541	0.8847	0.0824	2.6758
9	0.3521	5.2921	0.3037	0.2053	5.9317
10	0.2160	12.1630	0.0244	0.1081	3.5001
11	0.2569	11.2379	0.5773	0.1471	5.1637
12	0.1886	25.2275	0.5691	0.0870	3.0455
Corr($\tilde \nu $,.)	0.9662	− 0.6486	− 0.1934	0.9755	1.0000
*p*-value	0.0000	0.0225	0.5469	0.0000	1.0000

We use an intercept *C* in all the specifications. The first column in Panel A represents the number of cases and the second one the maximum number of parameters *p* in the regression model. When both *W* *X* and a time lag are included in the model, this means that *Wy*_1990_ is also added. This represents the last row in Panel A where 12 cases are created with a number of estimated parameters *p* varying between one and 26. Because the log-price in 2000 *P*_2*K*_ is regressed on variables from 1990, it indirectly includes various macro factors such as inflation. However, any adjustment for inflation made over the entire sample would proportionately affect all the estimated *β* coefficients and variance. It would not affect the kurtosis of the residuals, the focus of this exercise, since multiplicative transformations do not affect the kurtosis of a random variable.

Panel B in Table 10 shows the standard deviation of the residuals, estimated kurtosis, skewness, median absolute errors and the estimated degrees-of-freedom $\tilde \nu $ from a Student *t* distribution fitted to the residuals. The intercept only model (Case= 1) shows mild levels of kurtosis (3.55) and skewness with a high amount of error in the residuals (median absolute error of 39.2%). The estimated degrees-of-freedom $\tilde \nu $ is 12.37. The addition of spatial, temporal and explanatory variables (Case= 12) reduce the median absolute error to 8.7% but raise the estimated kurtosis to 25.23, and decrease the estimated degrees-of-freedom $\tilde \nu $ to 3.05. The correlation between the median absolute error and the estimated degrees of freedom $\tilde \nu $ is 0.976.

**Fig. 1:**
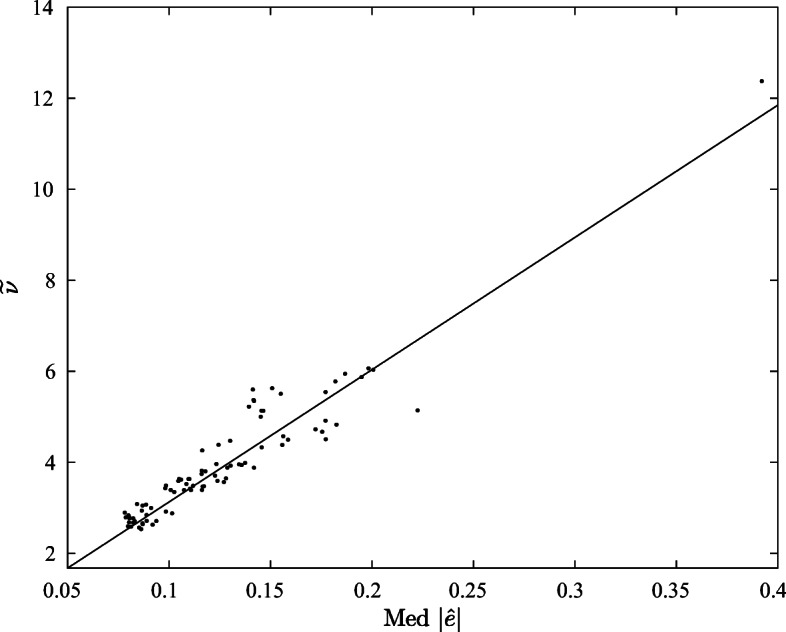
Relation between estimated degrees-of-freedom $\tilde \nu $ and median absolute errors med |*e*|

We plot in Figure 1 the relation between the estimated median absolute residuals |*e*| and the estimated degrees-of-freedom $\tilde \nu $.

In addition to the results reported in Table 10, we consider the following additional specifications that might affect the fit:

- squares of the explanatory variables
- both *WX* and *W*^2^*X*
- contiguity as well as 15 and 30 nearest neighbor *W*
- matrix exponential spatial specification for autoregressive *y* as well as spatial fractional differencing for autoregressive *y* (LeSage and Pace 2007; 2009; Debarsy et al. 2015). The matrix exponential spatial specification falls between MA and AR specifications for lower-order spatial lags while the fractional differencing assigns more importance to higher-order spatial lags than AR. Higher order ARMA models can approximate, albeit with more parameters, fractional differencing models (Haubrich 1993, p. 767).

In total, this led to 88 different specifications attempting to capture temporal dependence, non-linearities in the response, and many forms of spatial dependence in the dependent and independent variables. For all these specifications, we still obtain that the residuals demonstrate a striking relation between goodness-of-fit and measured levels of leptokurtosis ($\tilde \nu $). This is confirmed by the high correlation (0.9630) between the median absolute error and the estimated degrees of freedom $\tilde \nu $.

## Conclusion

In a spatial regression model, non-normality of the underlying independent disturbances has little effect on the accuracy of the parameter estimates and on the point prediction of the dependent variable for large samples (Pace and LeSage 2008). However, it does matter greatly for prediction intervals as they depend on the distribution of the underlying disturbances as well as the distribution of the prediction which will exhibit a low variance for large *n*. The coverage of the prediction interval is important for many applications such as a house price stress test (Follain and Giertz 2011). An important goal for many of these applications is to accurately model extreme (rare) values, also known as black swans.

Dependence in a spatial error model means that observed residuals come from weighted sums of underlying independent innovations. If the underlying innovations are normally distributed, the observed residuals should be normal as well. However, if the underlying innovations are non-normal, the resulting weighted sums can appear more normal than the underlying innovations. In such cases, in this paper we show theoretically, on Monte Carlo simulations and empirical data that ignoring spatial or spatiotemporal dependence in the disturbances can make the distribution of residuals appear more normal than the underlying innovations. As this can lead to inaccurate estimates for the extreme (rare) values, the fog of spatial and temporal dependence can make black swans appear grey.

## Footnotes

1 Carbone and Longini (1977) examine some characteristics an AVM should exhibit to meet public acceptability and efficacy criteria.

2 Zillow uses their estimates as part of their process to buy houses for resale and so they have a direct incentive to perform well (Duffy 2021).

3 See LeSage and Pace (2009) for more details on the construction of *W*.

4 If *W* is a sparse matrix, finding the solution for ***x*** of this problem is typically very fast.
